# Anxiety and Depression in the Relatives of COVID-19 and Non-COVID-19 Intensive Care Patients During the Pandemic

**DOI:** 10.7759/cureus.20559

**Published:** 2021-12-21

**Authors:** Behiye Deniz Kosovali, Busra Tezcan, Ismail Aytaç, Tulay Tuncer Peker, Ozlem B Soyal, Nevzat Mehmet Mutlu

**Affiliations:** 1 Department of Critical Care Medicine, Health of Science Ankara City Hospital, Ankara, TUR; 2 Department of Anesthesiology and Reanimation, Health of Science Ankara City Hospial, Ankara, TUR; 3 Department of Critical Care Medicine, Gulhane Education and Research Hospital, Ankara, TUR; 4 Department of Anesthesiology and Reanimation, Health of Science Ankara City Hospital, Ankara, TUR

**Keywords:** anxiety level, depression, relative, intensive care unit, hospital anxiety depression scale (hads), covid-19

## Abstract

Background and aim

In the literature, there is no study on the anxiety and depression status of the relatives of intensive care COVID-19 and non-COVID-19 patients during the pandemic period. In this study, we aimed to compare the risk of developing anxiety and depression in the relatives of COVID-19 and non-COVID-19 intensive care patients during the pandemic, and also to determine the factors that may cause anxiety and depression.

Materials and methods

Relatives of patients admitted to Ankara City Hospital COVID-19 (n=45) and non-COVID-19 (n=45) intensive care units between 15 May and 31 July 2021 were included in this prospective study. The Hospital Anxiety and Depression Scale (HADS) questionnaire was administered to the relatives of the patients within the first 48 hours of their admission to the intensive care unit. The answers were recorded and HADS, HADS-A (anxiety) and HADS-D (depression) scores were calculated accordingly. Demographics, education and marital statuses of both the patients and their relatives were recorded. Logistic regression analysis was performed to determine the factors associated with depression and anxiety. Receiver operator characteristics (ROC) curves were drawn for the factors affecting depression and anxiety, and the area under the curve values were calculated.

Results

Demographics, APACHE II score, and patient affiliation were similar in both groups. The mean HADS scores of the relatives of COVID-19 and non-COVID-19 patients were 24.76 and 16.04 (p<0.001). The mean HADS-A scores were 12.89 and 7.78 (p<0.001), and the mean of HADS-D scores were 11.87 and 8.27 (p=0.001). Moderate and high-risk anxiety and depression were significantly higher in relatives of COVID-19 patients (p=0.018, p=0.001, respectively). The area under curve (AUC) values were 0.727 in the ROC curve plotted for the independent risk factor Q3 responses that reduced anxiety, and 0.791 and 0.785 in the ROC curve drawn for the independent risk factor Q1 and Q3 responses that reduced the development of depression.

Conclusion

We found that the anxiety and depression risk of the relatives of COVID-19 patients in the intensive care unit during the pandemic period is significantly higher than the relatives of non-COVID-19 patients in the intensive care unit. In addition, regardless of the diagnosis, younger intensive care patients may increase the anxiety and depression of the relatives of the patients during the pandemic. The higher-education level of the relatives of patients was determined as a factor reducing anxiety and depression.

## Introduction

On March 11, 2020, when the World Health Organization (WHO) declared a pandemic, the first case of coronavirus disease 2019 (COVID-19) in our country was reported by the Ministry of Health [1]. Within the scope of protective measures against COVID-19, which is very contagious and easy to treat, and whose treatment is still unknown, isolation, restrictions and pandemic rules were applied in Turkey as well as all over the world. These obligations necessitated some changes in people's lifestyle, working order and social life. All these measures may have created anxiety, stress, tension and depression in society. Pandemic diseases caused by infectious diseases have negative effects not only on physical health but also on mental health [2]. It is known that there is an increase in the prevalence of psychiatric symptoms such as post-traumatic stress disorder, major depression, anxiety and sleep disorders after natural or man-made disasters [3]. During the COVID-19 pandemic, individuals may carry the anxiety, fear, and stress of getting the disease or infecting another person. In addition, the mortality of the disease and the large number of casualties all over the world can increase mental stress and trauma.

With the declaration of the COVID-19 pandemic, the world focused on this disease and the agenda was and still is COVID-19. However, during this period, other acute or chronic diseases (such as hypertension, diabetes mellitus, chronic obstructive pulmonary disease, myocardial infarction, acute stroke, cancer, trauma) other than COVID-19 did not disappear. During the pandemic period, there were hospitalizations and even intensive care unit (ICU) admissions not only due to COVID-19 but also to other diseases. Due to the airborne transmission in COVID-19, the visiting restrictions in hospitals in Turkey were implemented in both COVID-19 and non-COVID-19 inpatient services and ICU. Thus, direct communication of patients with their relatives was restricted. The relatives of the patients were informed by the doctors at regular intervals by phone. Although there is a study in the literature regarding anxiety and depression of relatives of patients who were excluded from the diagnosis of COVID-19 after being admitted to the ICU with a pre-diagnosis of COVID-19, there was no study evaluating the anxiety and depression of relatives of patients in COVID-19 and non-COVID-19 ICUs.

The aim of this study is to compare the risk of developing anxiety and depression in the relatives of COVID-19 vs non-COVID-19 intensive care patients during the pandemic period. It is also to determine the factors that can cause anxiety and depression.

## Materials and methods

The study was conducted prospectively with the relatives of the patients who were admitted to the Ankara City Hospital COVID-19 and non-COVID-19 ICUs between 15 May and 31 July 2021, after the approval of the ethics committee (Ethics committee approval number: E1-20-526).

The 18 years and older relatives of 90 ICU patients, half of whom were hospitalized in the COVID-19 ICU with positive polymerase chain reaction (PCR) test in nasopharyngeal or deep tracheal aspirate samples (n=45), and the other half who hospitalized in the non-COVID-19 ICU due to non-COVID-19 causes after exclusion of COVID-19 clinically, radiologically as well as with a negative PCR test (n=45) were included in the study. During the pandemic, the consent of the relatives of the patients included in the study was taken verbally during the phone interview due to the restrictions for the relatives of the patients to enter the hospital by the Ministry of Health. While informing the relatives of the patients by phone, the "Hospital Anxiety and Depression Scale (HADS)" questionnaire was applied to the relatives of the patients within the first 48 hours of their admission to the ICU by the intensive care doctor who followed the patient and informed the patient's relatives. Before the HADS questionnaire, five different questions with four options between 0-3 points were asked to the relatives of the patients. Patient visits were restricted in the pandemic. Patients' relatives and physicians could not meet face-to-face and patient relatives were informed by phone. These questions were asked to measure the risk of anxiety and depression of patients' relatives due to these reasons (Table 1).

**Table 1:** Questions evaluating the cause of anxiety and depression

Questions evaluating the cause of anxiety and depression
Question 1. How concerning is your patient’s hospitalization in the ICU due to an epidemic?
0) Not at all 1) Sometimes 2) Very often 3) Most of the time
Question 2. How much are you concerned about getting sick from your patient?
0) Not at all 1) Sometimes 2) Very often 3) Most of the time
Question 3. How concerning is it not being able to visit your patient in the ICU?
0) Not at all 1) Sometimes 2) Very often 3) Most of the time
Question 4. How concerning is the way you receive information about your patient (over the phone)?
0) Not at all 1) Sometimes 2) Very often 3) Most of the time
Question 5. How concerning is the frequency of getting information about your patient (3 times a week)?
0) Not at all 1) Sometimes 2) Very often 3) Most of the time

Participants with previous or ongoing psychiatric illness as well as the ones who refused to participate in the study or cannot communicate and cooperate enough to complete the surveys during a phone call and younger than 18 years of age were excluded from the study.

The age, gender, education level (primary school, high school, university, illiterate), marital status (married, single, divorced, widowed) of the patients and their relatives were recorded. Acute Physiology and Chronic Health Evaluation II (APACHE-II) scores of the patients were recorded in the first 24 hours. According to the WHO age classification, the patients were divided into three groups as 18-65 years old, 66-80 years old and 81-99 years old, and into five groups as spouse, child, parent, sibling and next of kin in terms of closeness of patients with their relatives [4]. Relatives of patients were grouped as private sector employees, civil servants, retired, unemployed and students according to their occupational characteristics.

The HADS questionnaire, which was defined by Zigmond and Snaith and translated into Turkish by Aydemir et al. in Turkey and validated for the Turkish population, was used to measure the anxiety and depression levels of patients' relatives [5,6]. HADS consists of 14 questions, each question consists of four options with a value between 0-3 points. The general HADS score with the total score of all questions (0-42 points), anxiety score with seven odd-numbered questions (HADS-A) (0-21 points), depression score with seven even-numbered questions (HADS-D) (0 -21 points) were calculated. The HADS questionnaire was applied to the relatives of the patients on the phone and the answers were recorded and the score was calculated duly. According to the validation of the HADS questionnaire for the Turkish population, cut-off values were accepted as >10 for anxiety and >7 for depression [6]. HADS-A (0-10 normal, 11-15 moderate risk of anxiety, 16-21 high risk of anxiety) and HADS-D (0-7 normal, 8-10 moderate risk of depression, 11-21 high risk of depression), each of which is divided into three subgroups, were evaluated.

Statistical analysis

Statistical analyses of the data obtained in the study were performed using the SPSS for Windows 26.0 statistical analysis software (IBM Corp, Armonk, USA). Continuous variables were expressed as mean±SD. After evaluating the conformity of numerical data to normal distribution with the Shapiro-Wilk test, Student's t-test was used to compare the numerical data with normal distribution, and the result was evaluated according to the equality of variances. Mann-Whitney U was used to compare the numerical data that were not normally distributed. Categorical data were given as numbers and percentages. Pearson Chi-Square test was used to compare categorical data. Logistic regression analysis was performed to determine the factors associated with depression and anxiety. The area under the curve (AUC) values were calculated by drawing ROC curves for the effective factors. P<0.05 was considered significant. Post hoc power analysis of the study was calculated with G*Power; the power of the study was determined 74%. 

## Results

The mean ages of COVID-19 and non-COVID-19 patients were 66.96 and 68.91 years, respectively (p=0.560). Male gender ratios were 51.1% and 53.3% (p=0.833). While there were differences between the patient groups in terms of marital status (p=0.001), education levels, patient closeness and APACHE II score averages of patients were similar in both groups (p=0.209, p=0.548, p=0.099, respectively). The mean ages of COVID-19 patient relatives and non-COVID-19 patient relatives were 42.73 and 48.6 years, respectively, and there was statistical significance (p=0.026), however, the gender distribution was similar (p=0.130). The marital status, education level and occupational distribution of the relatives of the patients in both groups were similar (p=0.522, p=0.347, p=0.603 respectively) (Table 2).

**Table 2:** Demographic characteristics of patients and patients’ relatives APACHE II Score: Acute Physiology And Chronic Health Evaluation Score

Variables	COVID-19 Patients n=45	Non-COVID- 19 Patients n=45	p	COVID-19 Patients’ relatives n=45	Non-COVID- 19 Patients’ relatives n=45	p
Age (Mean±SD)	66.96±12.7	68.91±18.5	0.560	42.73±12.4	48.6±12.2	0.026
Female	70.86±10.1	77.05±11.4		42.77±12.9	50.38±12.9	
Male	63.33±13.9	61.79±20.6		42.7±12.1	47.04±11.6	
Female n(%)	22 (48.9)	21 (46.7)	0.833	14 (31.1)	21 (46.7)	0.130
Male n(%)	23 (51.1)	24 (53.3)		31 (68.9)	24 (53.3)	
Marital status n(%)	40 (88.9)	23 (51.1)	0.001	37 (82.2)	38 (84.4)	0.522
Married						
Single	1 (2.2)	6 (13.3)		8 (17.8)	6 (13.3)	
Divorced	0	1 (2.2)		0	0	
Widow	4 (8.9)	15 (33.3)		0	1 (2.2)	
Education n(%)	25 (55.6)	32 (71.1)	0.209	8 (17.8)	13 (28.9)	0.347
Primary school						
High school	6 (13.3)	7 (15.6)		16 (35.6)	11 (24.4)	
University	7 (15.6)	2 (4.4)		21 (46.7)	21 (46.7)	
Illiterate	7 (15.6)	4 (8.9)		0	0	
Degree of Kinship n(%) Spouse	5 (11.1)	5 (11.1)	0.548	-	-	-
Child	32 (71.1)	25 (55.6)				
Relative	5 (11.1)	10 (22.2)				
Sibling	2 (4.4)	3 (6.7)				
Parent	1 (2.2)	2 (4.4)				
Profession n(%)	-	-	-	22 (48.9)	19 (42.2)	0.603
Private employee						
Civil servant				8 (17.8)	10 (22.2)	
Retired				4 (8.9)	4 (8.9)	
Unemployed				8 (17.8)	5 (11.1)	
Student				3 (6.7)	7 (15.6)	
APACHE II Score (Mean±SD)	16.56±7.1	19.13±7.6	0.099	-	-	-

The mean HADS values of the relatives of COVID-19 and non-COVID-19 patients were 24.76, 16.04, anxiety (HADS-A) mean was 12.89, 7.78, and depression (HADS-D) mean was 11.87, 8.27 (p<0.001, p<0.001, p=0.001), respectively. When anxiety and depression risks were classified, the risk of moderate and high anxiety and depression was significantly higher in the relatives of COVID-19 patients (p=0.018, p=0.001, respectively) (Table 3).

**Table 3:** Comparison of the COVID-19 and nonCOVID-19 patients relatives’ HADS survey results HADS: Hospital anxiety and depression scale, HADS-A score: Hospital anxiety and depression scale-Anxiety score, HADS-D score: Hospital anxiety and depression scale-Depression score

Variables	COVID-19 Patients’ relatives	Non-COVID-19 Patients’ relatives	p
HADS (Mean±SD)	24.76±8.8	16.04±11.6	<0.001
HADS-A score (Mean±SD)	12.89±4.9	7.78±6.1	<0.001
0-10 n(%)	15 (33.3)	28 (62.2)	0.018
11-15 n(%)	16 (35.6)	11 (24.4)	
16-21 n(%)	14 (31.1)	6 (13.3)	
HADS-D score (Mean±SD)	11.87±4.5	8.27±5.8	0.001
0-7 n(%)	6 (13.3)	22 (48.9)	0.001
8-10 n(%)	13 (28.9)	8 (17.8)	
11-21 n,(%)	26 (57.8)	15 (33.3)	

The HADS-A, HADS-D and HADS values were compared in Table 4 according to the level of kinship in both groups with the patient, age classification of the patients, and education level of the relatives of the patients.

**Table 4:** HADS, HADS-A and HADS-D according to level of kinship, age classification, education level HADS-A score: Hospital anxiety depression scale-Anxiety score, HADS-D sore: Hospital anxiety depression scale-Depression score, HADS: Hospital anxiety depression scale (total score)

Variables	HADS-A score		p	HADS-D score		p	HADS		p
	COVID-19 Patients	Non-COVID- 19 Patients		COVID-19 Patients	Non-COVID -19 Patients		COVID-19 Patients	Non-COVID -19 Patients	
Spouse	15.6±6.1	8.8±7.4	0.151	14.4±7.2	8.6±5.7	0.195	30±13.2	17.4±13.1	0.167
Child	12.19±4.8	7±6.1	0.001	11.03±3.9	7.44±5.8	0.011	23.22±7.7	14.44±11.6	0.002
Relative	15.4±5.1	6.5±5.5	0.010	14.6±5.4	7±5.3	0.022	30±10.2	13.5±10.4	0.012
Sibling	10±1.4	10.67±2.3	0.746	11±2.8	12.67±2.1	0.495	21±4.2	23.33±4.1	0.578
Parents	15	17±2.8	0.667	14	17.5±0.7	0.154	29	34.5±2.1	0.281
18-65 age	12.83±4.5	11.82±5.2	0.566	11.13±4.3	12.18±4.8	0.527	23.96±8.1	24±9.6	0.989
66-80 age	14±5.5	8.21±6.5	0.014	14±4.9	8.58±6	0.011	28±9.8	16.79±12.2	0.010
81-99 age	11.44±5.1	4.27±4.1	0.001	10.67±3.8	5±4.3	0.003	22.11±8.7	9.27±8.2	0.001
Primary school	13.38±6.5	6.62±7.2	0.043	12.38±5.5	7.23±6.6	0.082	25.75±11.9	13.85±13.7	0.057
High school	13.06±3.4	8.36±5.7	0.012	12.5±4	9±5.6	0.068	25.56±6.7	17.36±10.8	0.022
University	12.57±5.4	8.19±5.8	0.016	11.19±4.7	8.52±5.5	0.098	23.76±9.2	16.71±11	0.030

In order to determine the risk factors for anxiety and depression in all patients' relatives, the patient's age, the education level of the patient's relatives, and the response values to five questions were included in the logistic model as explanatory independent variables.

While the age of the patient was an independent risk factor that increased the development of anxiety, Q3 responses and the education level of the patient's relatives were determined as the factors that decreased the development of anxiety (OR: 1.060, 0.472, 0.170, respectively) (Table 5).

**Table 5:** Multivariate logistic regression analysis for anxiety SE: Standart Error, Q1: Question 1, Q2: Question 2, Q3: Question 3, Q4: Question 4, Q5: Question 5

Variable	B (Coefficient)	SE	Confidence Interval	Odds Ratio	p
Constant	-0.419	1.350		0.657	0.756
Patient age	0.058	0.020	1.019-1.103	1.060	0.004
Q1	-0.682	0.360	0.250-1.024	0.506	0.058
Q2	-0.103	0.285	0.516-1.577	0.902	0.717
Q3	-0.751	0.395	0.218-1.023	0.472	0.024
Q4	0.110	0.345	0.568-2.193	1.116	0.750
Q5	-0.190	0.361	0.408-1.677	0.827	0.599
Education level of the patient's relative	-1.771	0.748	0.039-0.736	0.170	0.018
R2: 0.426 (Cox-Snell), R2: 0.599 (Nagelkerke)					

The age of the patient was also found to be an independent risk factor that increased the development of depression, while the Q1 and Q3 responses and the education level of the patient's relatives was an independent factor that decreased the incidence of depression (OR: 1.084, 0.352, 0.486, 0.173, respectively) (Table 6).

**Table 6:** Multivariate logistic regression analysis for depression SE: Standart Error, Q1: Question 1, Q2: Question 2, Q3: Question 3, Q4: Question 4, Q5: Question 5

Variable	B (Coefficient)	SE	Confidence Interval	Odds Ratio	p
Constant	-2.943	1.946		0.053	0.130
Patient age	0.80	0.027	1.027-1.143	1.084	0.003
Q1	-1.045	0.454	0.144-0.857	0.352	0.021
Q2	-0.012	0.392	0.458-2.132	0.988	0.975
Q3	-0.978	0.432	0.161-0.877	0.486	0.024
Q4	0.137	0.474	0.453-2.904	1.147	0.772
Q5	0.205	0.498	0.463-3.257	1.228	0.680
Education level of the patient's relative	-1.756	0,865	0.032-0.940	0.173	0.042
R2: 0.361 (Cox-Snell), R2: 0.481 (Nagelkerke)					

**Figure 1:**
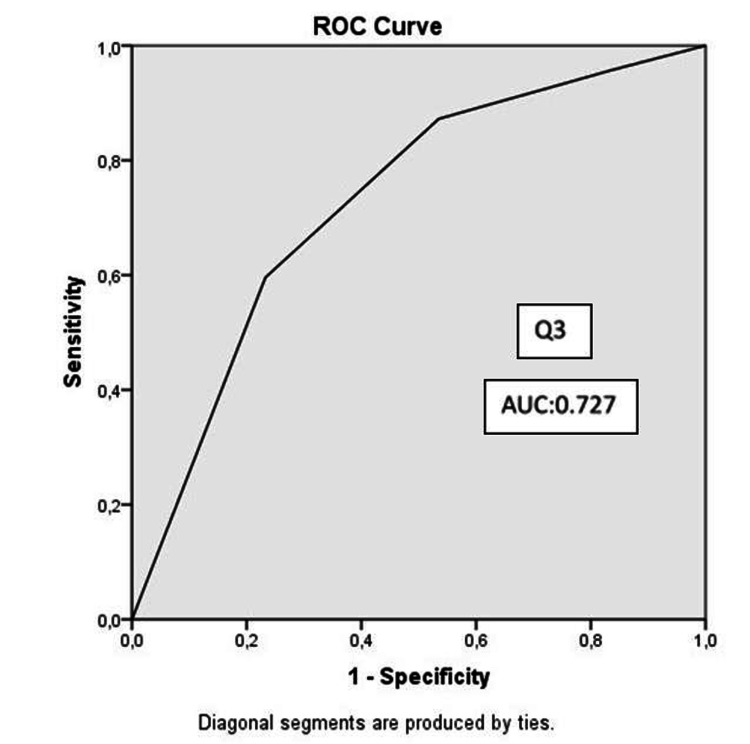
ROC curve for the model predicting anxiety Q3: Question 3, AUC: area under the curve, ROC: reciever operator characteristics

**Figure 2:**
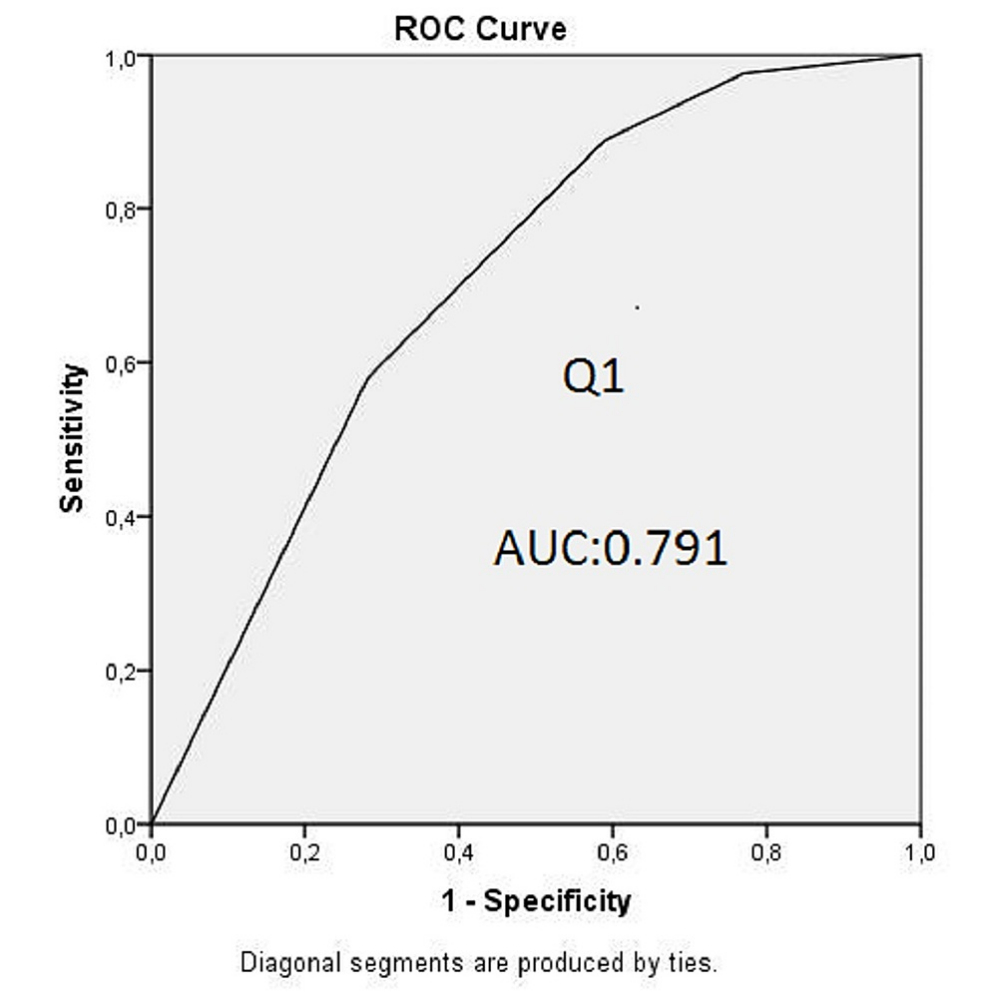
ROC curve for the model predicting depression (Q1) Q1: Question 1, AUC: area under the curve, ROC: reciever operator characteristics

**Figure 3:**
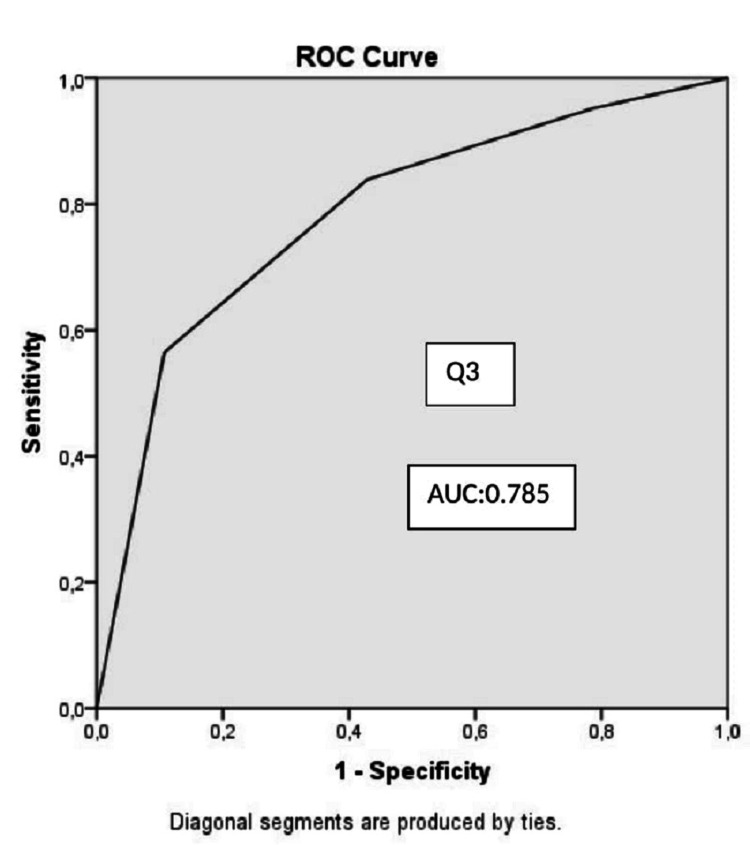
ROC curve for the model predicting depression (Q3) Q3: Question 3, AUC: area under the curve, ROC: reciever operator characteristics

AUC values were 0.727 in the ROC curve drawn for responses to the independent risk factor Q3 that reduced anxiety, and 0.791 and 0.785 in the ROC curve that was drawn for responses to the independent risk factor Q1 and Q3 that reduced the development of depression, respectively (Figures 1, 2, 3).

## Discussion

Along with the COVID-19 pandemic, various studies have been reported in the literature measuring the anxiety and depression frequency of COVID-19 patients and/or their relatives or referring to their experiences [7,8,9,10]. In addition, in a previous study, we reported by measuring the anxiety and depression levels of the relatives of patients who were followed up in the intensive care unit with the suspicion of COVID-19 during the pandemic [11]. In this study the relatives were divided into two groups according to the diagnosis of their patients as COVID-19 or non-COVID-19. It was aimed to determine the change in anxiety and depression scores of the relatives of the patients after the diagnosis and suspicion of COVID-19 by applying the HADS questionnaire twice, before and after the diagnosis of COVID-19 became clear to the relatives of the patients [11]. This was a prospective observational cohort study held in Turkey, to evaluate the anxiety and depression frequency as well as potential risk factors concerning the relatives of the patients who were followed up in the COVID-19 and non-COVID-19 intensive care units with or without a positive COVID-19 PCR test during the pandemic.

Köse et al. showed the anxiety and depression levels of relatives of intensive care patients in Turkey before the pandemic. The rates of anxiety and depression were reported as 35.9% and 71.8%, respectively [12]. In another study, the anxiety and depression rates of the general population in Turkey during the pandemic period were reported as 45.1% and 23.6%, respectively [13]. Again, in the study reported by Kosovalı et al during the pandemic in Turkey, the anxiety rate of the relatives of patients with COVID-19 was reported as 66.6% and the depression rate as 71.7% [11]. In the present study, the anxiety rate (66.7%) of the relatives of COVID-19 patients was similar, while the depression rate (86.7%) was higher than the results of the studies in the literature [11,12,13]. Considering the results of this study, which was also conducted in the same country during the pandemic period, we can say that the anxiety rate of the relatives of COVID-19 patients did not decrease during the pandemic period, and that the rates of depression increased over time [11]. Since the study by Kosovalı et al. was conducted during the first wave of the pandemic, patient relatives may have hoped that the disease would end in a short time, that treatment would be found, or that the severity of the disease would decrease over time [11]. In this study, which was conducted during the second wave of the pandemic, we can explain the increase in the frequency of anxiety and depression when the life-limiting factors such as isolation and quarantine were added to the negativities such as the ongoing disease, the death of hundreds of people every day in the country and the world, the lack of treatment, the uncertainty of the effectiveness of the vaccine. In a study reported from the United States, the frequency of depression increased three times during the pandemic period compared to the pre-pandemic period, and in another study conducted in Switzerland, an increase in the stress level and symptoms of depression was observed in each new pandemic wave [14,15]. Similar results were obtained in the present study. Even though countries and cultures may change, we can say that the level of anxiety and depression in all societies has increased gradually during the pandemic.

In the present study, the rate of anxiety risk (37.7%) in the relatives of non-COVID-19 patients was higher than the anxiety rate (35.9%) of intensive care patients in a study conducted in the pre-pandemic period, and lower than the depression rate (71.8% vs 51.1%) [12]. When the anxiety level of the relatives of the non-COVID-19 patients in the study conducted during the first wave of the pandemic was compared, we can say that the anxiety level of the relatives of the non-COVID-19 patients in the present study was higher (26.7% vs 37.7%) while the depression level was similar (53.3% vs 51.1%) [11]. In the study of Kosovalı et al, anxiety and depression scales were measured twice. The first measurement was made before the diagnosis of COVID-19, and the second measurements were made after the diagnosis of COVID-19 became clear. It should be considered that the anxiety level of the relatives of the patients in the non-COVID-19 group is the level of anxiety after learning that their patients are negative for coronavirus.

The average HADS of the relatives of COVID-19 patients in the ICU was significantly higher than the relatives of non-COVID-19 patients. Also, the rate of moderate and high anxiety and depression risk in the classification according to HADS-A and HADS-D scores was significantly higher in the relatives of COVID-19 patients. With all this data, although being a relative of an intensive care patient is an independent risk factor for anxiety and depression, we can say that being a relative of a patient hospitalized in an ICU with a diagnosis of COVID-19 higher risk factor for anxiety and depression.

Although there was no significant difference between the HADS-A, HADS-D and HADS averages of the spouses of COVID-19 and non-COVID-19 patients in this study, these values were higher in the spouses of COVID-19 patients. In the previous study of Kose et al., similar to the results of our study, it was reported that the frequency of anxiety and depression was high in the spouses and children of the patients [12]. As a spouse sharing a common life at the time, as children who know that their parents are in intensive care, the need for intensive care that develops unexpectedly can increase anxiety and depression. In addition, having to struggle with a disease that the world is affected by and the negativities of life affected by this disease, not being able to see their relatives in intensive care, not being with them, fear of losing the person they love may increase the anxiety and depression of individuals. Considering the high probability of domestic transmission in COVID-19, the person may be in quarantine at home while his wife is treated in the intensive care unit. This can make individuals even more anxious. A similar situation also counts for other family members [11].

Many studies have shown that mortality in advanced age increases in COVID-19 [16,17]. The widespread sharing of this situation on social media may have made the relatives of the patients aware of the increase in mortality in elderly patients. In this study, the mean anxiety and depression scores were higher in the relatives of COVID-19 patients aged >65 years than the non-COVID-19 group. This may be due to the fear of losing their elderly patients. 

For all patients' relatives, being young in ICU was an independent risk factor that increased anxiety and depression. This result was similar to the results of previous studies [11,18,19]. The fact that the patient treated in the ICU is young and needs support systems such as mechanical ventilators rather than being diagnosed with COVID-19 or non-COVID-19 is a traumatic process for his relatives and may increase stress. This serious illness, which develops at an unexpected time, can leave families in a difficult situation financially as well as psychologically. Mortality or morbidity of a young family member may cause anxiety and depression in other family members both socially and economically.

In the present study, being unable to visit a patient in the ICU was found to be a factor reducing anxiety in individuals with higher education levels. This may be due to the fact that individuals with a high level of education have better perceived that activities such as patient visits during the pandemic are factors that increase the risk of transmission. Similarly, the fact that the patient is hospitalized during the pandemic period creates the impression that it will increase the tendency to depression. Contrary to the previous study, in this study, visit restriction and the fact that the patient was hospitalized in the ICU during the pandemic were found to be factors that reduce the level of anxiety and depression in individuals with a high level of education [11]. The reason for this may be that he/she had the chance to be treated in a safe environment such as intensive care, since he/she was sick at home or could not accompany his/her patient due to the risk of transmission during the pandemic period. Because at the beginning of the pandemic, images of patients waiting to be treated on stretchers in the hospital corridors or gardens were frequently featured in the media. Therefore, even the fact that intensive care units were found in hospitals during the pandemic period when intensive care bed capacity was limited may have reduced these risk factors.

Limitations of the study are: the fact that it was single-centered, the sample size of participants included in the study was small, and a questionnaire that did not diagnose anxiety and depression but measured the risk of developing anxiety and depression by scoring method was used.

## Conclusions

We found that anxiety and depression may be higher in the relatives of patients with COVID-19 who are followed up in the ICU, regardless of the diagnosis, being young in all intensive care patients may increase the anxiety and depression of the relatives of the patients during the pandemic period. Higher education level of the relatives of the patient may reduce anxiety and depression.
